# Preharvest Application of Exogenous 2,4-Epibrassinolide and Melatonin Enhances the Maturity and Flue-Cured Quality of Tobacco Leaves

**DOI:** 10.3390/plants13233266

**Published:** 2024-11-21

**Authors:** Kesu Wei, Jiayi Tang, Lei Yang, Shaopeng Chen, Zhijun Cheng, Yijun Yang, Chen Xu, Shengjiang Wu, Yuhang Zhao, Hongmei Di, Ling Li, Dongyang Sun, Jianwei Li, Bo Sun

**Affiliations:** 1 Guizhou Academy of Tobacco Science, Guizhou Provincial Academician Workstation of Microbiology and Health, Guiyang 550081, China; weiks8816@163.com (K.W.); 2023205039@stu.sicau.edu.cn (J.T.); furongwang2003@126.com (L.Y.); chengzhj0619@hngytobacco.com (Z.C.); yangyj0518@hngytobacco.com (Y.Y.); wushengjiang1210@163.com (S.W.); txek123456789@126.com (Y.Z.); 2 College of Horticulture, Sichuan Agricultural University, Chengdu 611130, China; 2023105007@stu.sicau.edu.cn (H.D.); 2023205038@stu.sicau.edu.cn (L.L.); 2024305096@stu.sicau.edu.cn (D.S.); 3 Chongqing Tobacco Science Institute, Chongqing 409199, China; chenshaopeng3311@dingtalk.com (S.C.); cqxuchen@139.com (C.X.)

**Keywords:** *Nicotiana tabacum*, 2,4-epibrassinolide, melatonin, flue-cured quality, transcriptome, metabolome

## Abstract

Tobacco (*Nicotiana tabacum*) is a globally cultivated crop, with its quality closely associated with the color and chemical composition of cured tobacco leaves. In this experiment, the effects of spraying exogenous 2, 4-epibrassinolide (EBR) and melatonin (MT) on the development of tobacco leaves at maturity stage and the quality after curing were investigated. Both EBR and MT treatments significantly enhanced the appearance quality of tobacco leaves at the stem-drying stage. Following preharvest applications, the sugar-to-alkali ratio and potassium content increased, while the contents of starch, total alkaloids, and proteins decreased. The levels of conventional chemical components were improved, enhancing the overall coordination of the tobacco. Transcriptome analysis revealed that EBR treatment down-regulated the chlorophyll biosynthetic genes *hemA*, *MgPEC*, and *ChlD*, while up-regulating the chlorophyll degradation genes *CHL2*, *SGR*, and *PAOs*. Similarly, MT treatment down-regulated the chlorophyll biosynthetic genes *FC2* and *MgPEC* and up-regulated the degradation genes *CHL2* and *SGR*, thus promoting chlorophyll degradation. Furthermore, in the downstream carotenoid biosynthetic pathway, both EBR and MT treatments regulated abscisic acid-related genes, with *NCEDs* being up-regulated and *CYP707A1s* down-regulated, thereby promoting the leaf ripening. Metabolomics analysis indicated that EBR treatment primarily regulated alkaloids, terpenoids, and flavonoids, while MT treatment mainly affected flavonoids. Both treatments also reduced the accumulation of the harmful substance aristolochic acid B. Comprehensive evaluations of appearance quality, physiological parameters, transcriptome, and metabolomics analyses demonstrated that exogenous spraying of EBR and MT treatments improved the maturity and quality of cured tobacco leaves, with EBR treatment exhibiting a greater effect than MT treatment.

## 1. Introduction

Tobacco (*Nicotiana tabacum*) is one of the most widely cultivated crops in the world [1], with its quality closely associated with the color and chemical composition of its leaves, attracting significant attention [2]. During the tobacco curing process, chlorophyll content decreases rapidly while carotenoid levels increase, resulting in an attractive appearance. However, natural disasters or improper field management practices, such as insufficient light, heavy rainfall, or excessive nitrogen application, can lead to “regreening,” where mature yellow tobacco leaves revert to green [3]. This regreening process, marked by chlorophyll resynthesis, delays maturity and adversely affects internal quality. When regreened tobacco leaves are harvested for curing, the high chlorophyll content degrades slowly, leading to rapid water loss and increased difficulty in the curing process. Consequently, the cured leaves exhibit an undesirable floating green color, severely compromising both external appearance and internal quality [4,5]. The chemical composition of tobacco leaves post-curing is a critical factor affecting tobacco quality. Carbohydrates, which are the most abundant aroma precursors, determine the sweetness and flammability of tobacco [6,7]. An appropriate level of alkaloids contributes to physiological satisfaction [8], while chlorine and potassium levels influence flammability [9]. In addition, metabolomics studies have identified secondary metabolites, such as flavonoids, terpenoids, and alkaloids, as key determinants of tobacco quality [1,2]. In recent years, various exogenous hormone treatments have been implemented to enhance crop quality [10,11,12].

Brassinosteroids (BRs) are significant growth regulators that influence plant physiology and play an important role in crop growth, development, and responses to biotic and abiotic stresses [10,13]. 2,4-Epibrassinolide (EBR) is a commercially available synthetic analogue of BRs, noted for its safety [14], which is widely used in crop production [15,16]. BRs have been shown to regulate tomato quality and ethylene-mediated fruit ripening, promoting lycopene biosynthesis and inhibiting chlorophyll synthesis through the transcriptional regulation of *LePSY1* and *LeGLK2* [17]. Exogenous application of EBR has been found to enhance the antioxidant capacity of sweet corn and significantly induce the expression of sucrose transporter genes, thereby improving the quality of sweet corn both at harvest and postharvest stages [18]. Additionally, EBR has been found to increase sucrose and hexose accumulation by promoting the activity and transcription of enzymes related to energy and sucrose metabolism, thereby maintaining high ATP content and energy status [19].

Melatonin (N-acetyl-5-methoxytryptamine, MT) is an indoleamine [20]. It serves as a signaling molecule widely present in plants, contributing to numerous physiological functions such as seed development, root growth, fruit ripening, maturation, senescence, and responses to both biotic and abiotic stresses [21]. Currently, MT is extensively utilized in tobacco production, where it enhances stress resistance under conditions of ozone [22], cadmium [23,24], and elevated NO_2_ levels [25]. Exogenous MT promotes the initiation and development of tobacco root hairs and long stalk glandular trichomes, improves the secretion capabilities of these trichomes, enhances tobacco’s resistance to aphids, and mitigates cadmium toxicity in tobacco plants [23]. In addition, MT contributes to the size and synchrony of grape berries prior to coloration and alters the aromatic components of wines, imparting more fruity, spicy, and sweet notes [26].

At present, numerous studies have investigated the effects of exogenous growth regulators on tobacco growth. However, limited research exists on the application of exogenous EBR and MT sprays on leaves during the maturity stage to regulate the ripening process and enhance the quality post-curing. This study aims to mitigate the delayed maturity caused by adverse weather and suboptimal management practices during early tobacco growth, as well as to minimize potential late-stage weather-related damage, such as frost damage in early autumn, which can be detrimental to tobacco production. Here, two exogenous growth regulators, EBR and MT, were applied to tobacco leaves pre-harvest to assess their effects on leaf maturity and quality at the stem-drying stage. Transcriptomic and metabolomic analyses provide theoretical insights into the mechanisms by which EBR and MT treatments improve tobacco leaf quality at the stem-drying stage.

## 2. Results

### 2.1. Appearance Quality Evaluation

**Figure 1:**
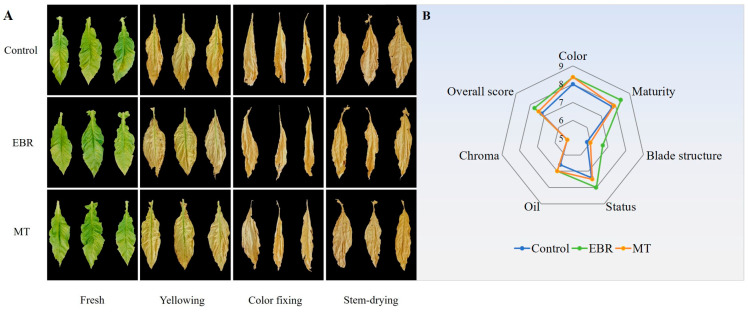
Tobacco during curing stage. (**A**) The appearance of tobacco during curing stage; (**B**) The appearance quality evaluation of tobacco leaves at stem-drying stage. EBR, tobacco leaves of EBR-treated; MT, tobacco leaves of MT-treated.

The appearance quality evaluation of tobacco leaves at the stem-drying stage served as a direct measure of their economic value (Figure 1A). The total scores for the three treatments were ranked as follows: EBR (7.71) > MT (7.42) > control (7.19). Notably, the maturity, blade structure, and status scores for the EBR treatment were the highest. Additionally, both EBR and MT treatments scored identically in color and oil content, with scores higher than the control (Figure 1B).

### 2.2. Chromatic Aberration

The fresh tobacco leaves treated with EBR appeared yellower, while those treated with MT and the control group appeared greener (Figure 1A). After EBR treatment, the a* of fresh tobacco leaves was significantly higher than that of the control, which was −7.66, and the b* was also significantly higher than that of the control, which was 38.79 (Supplementary Table S1).

No significant difference was observed in the a* value between the MT treatment and the control, whereas the b* value of the MT treatment was significantly lower than that of the control.

### 2.3. Chlorophyll and Carotenoids Content

**Figure 2:**
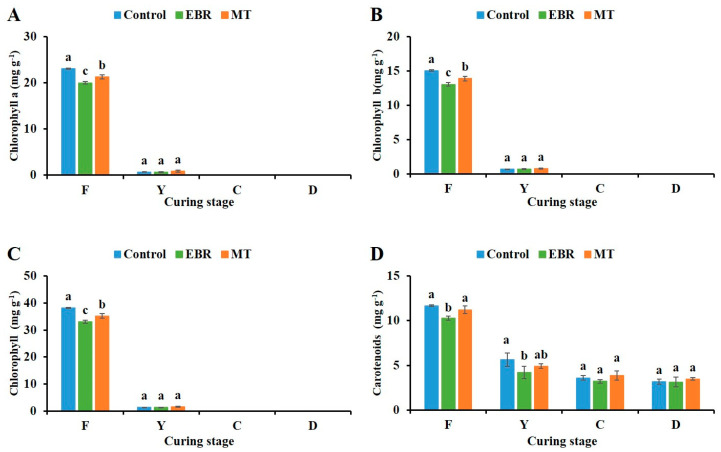
Pigment content of tobacco leaves during curing stage. (**A**) Chlorophyll a; (**B**) Chlorophyll b; (**C**) Chlorophyll; (**D**) Carotenoids. EBR, tobacco leaves of EBR-treated; MT, tobacco leaves of MT-treated; F, fresh leaves; Y, yellowing stage; C, color fixing stage; D, stem-drying stage. “a, b, c” in the table mean significant difference among different treatments (*p* < 0.05).

After EBR and MT treatments, the chlorophyll a, chlorophyll b, and total chlorophyll contents in fresh tobacco leaves were significantly lower than those in the control, with reductions of 5.05 and 2.91 mg g^−1^ DW for EBR and MT treatments, respectively (Figure 2A–C). After curing, the chlorophyll content in fresh tobacco leaves decreased sharply, with no significant differences observed among the treatment groups. At yellowing stage, chlorophyll degradation was nearly complete, leaving only trace amounts detectable. At both the color-fixing and stem-drying stages, chlorophyll was entirely undetectable.

In contrast to the control, the carotenoid content in fresh tobacco leaves treated with EBR was significantly lower, exhibiting a reduction of 11.84%. However, MT treatment did not have a significant effect on carotenoid levels (Figure 2D). Carotenoid content in fresh tobacco leaves began to decrease sharply during curing in the yellowing stage, with EBR-treated leaves containing only 74.45% of the carotenoid levels in the control. In the final two stages, no significant differences in carotenoid content were found among the EBR, MT, and control groups.

### 2.4. Moisture Content

**Figure 3:**
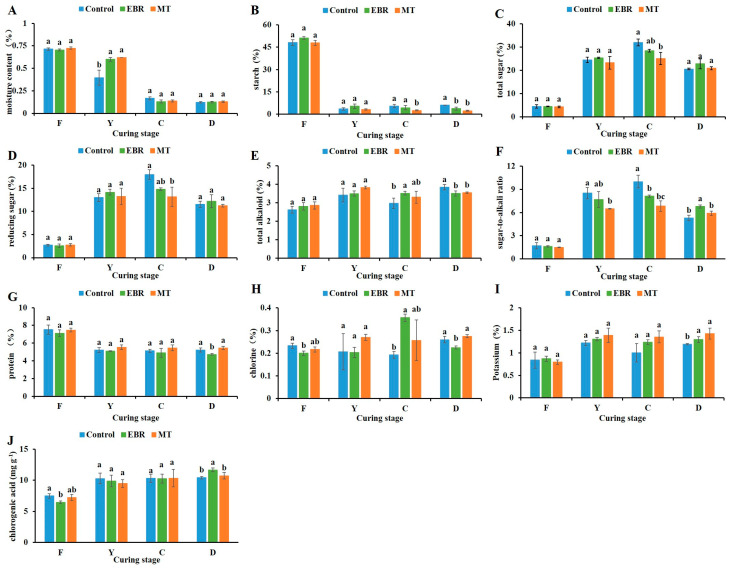
Physiological parameters of tobacco leaves during curing stage. (**A**) Moisture content; (**B**) Starch; (**C**) Total sugar; (**D**) Reducing sugar; (**E**) Total alkaloid; (**F**) Sugar-to-alkali ratio; (**G**) Protein; (**H**) Chlorinity; (**I**) Potassium; (**J**) Chlorogenic acid. EBR, tobacco leaves of EBR-treated; MT, tobacco leaves of MT-treated; F, fresh leaves; Y, yellowing stage; C, color fixing stage; D, stem-drying stage. “a, b” in the table mean significant difference among different treatments (*p* < 0.05).

The results showed that there was no significant difference in the moisture content of fresh tobacco leaves between EBR and MT treatments (Figure 3A). In control group, the moisture content of tobacco leaves decreased sharply in the ‘F–Y’ stage, whereas in the EBR and MT treatments, a sharp decrease occurred in the ‘Y–C’ stage. At the yellowing stage, the moisture content of tobacco leaves treated with EBR and MT was significantly higher than that of the control, at 1.51-fold and 1.56-fold the control, respectively.

### 2.5. Conventional Chemical Components

#### 2.5.1. Starch and Sugar

Compared to the control, EBR and MT treatments had no significant effect on the starch content of fresh tobacco leaves (Figure 3B). However, both treatments influenced the starch content during the late curing stage. Initially, the starch content of the tobacco leaves sharply decreased before stabilizing. Notably, at the stem-drying stage, the starch content in the EBR and MT treatments was significantly lower than in the control, with EBR-treated leaves showing a 39.87% reduction and MT-treated leaves showing a 62.27% reduction During the curing stage, the levels of total sugars and reducing sugars in tobacco leaves initially increased before subsequently decreasing. The content of total sugar and reducing sugar in MT treatment group was significantly lower than that in the control in the color fixing stage. In the ‘C–D’ stage, the content of total sugar and reducing sugar in tobacco leaves decreased across all groups (Figure 3C,D).

#### 2.5.2. Total Alkaloid

Compared to the control, EBR and MT treatments had no significant effect on the total alkaloid content of fresh tobacco leaves (Figure 3E). However, the total alkaloid content in the leaves began to increase after curing, with levels at the stem-drying stage being significantly lower for the EBR and MT treatments than for the control. Specifically, the total alkaloid content was reduced by 8.80% and 7.89% for the EBR and MT treatments, respectively.

#### 2.5.3. Sugar-to-Alkali Ratio

During the curing stage of tobacco leaves, the sugar-to-alkali ratio initially increased and then decreased (Figure 3F). At the color-fixing stage, the sugar-to-alkali ratios in the EBR and MT treatments were significantly lower than the control. The sugar-to-alkali ratio of EBR and MT treatments was significantly lower than the control at color fixing stage, and the Sugar-to-alkali ratio of tobacco leaves decreased sharply in the ‘C–D’ stage. The sugar-to-alkali ratio of tobacco leaves at stem-drying stage treated with EBR was significantly higher than the control, which was 1.28-fold the control. But there was no significant difference between MT treatment and the control.

#### 2.5.4. Protein

In contrast to the control, treatments with EBR and MT did not significantly affect the protein content of fresh tobacco leaves. However, the EBR treatment reduced the protein content of tobacco leaves at the stem-drying stage (Figure 3G). During the curing stage, the protein content of the leaves initially decreased and then stabilized. The protein content of tobacco leaves at the stem-drying stage treated with EBR was significantly lower than the control, exhibiting a reduction of 9.48%. In contrast, no significant difference was observed between the MT treatment and the control.

#### 2.5.5. Chlorine and Potassium

The chlorine content of fresh tobacco leaves was significantly decreased after EBR treatment (Figure 3H). At color fixing stage, the chlorine content of tobacco leaves treated with EBR was significantly higher than the control. In the ‘C–D’ stage, the chlorine content of tobacco leaves treated with EBR, while the control increased. The chlorine content of tobacco leaves at stem-drying stage treated with EBR was significantly lower than control, and its content was 13.46% lower than the control.

Both EBR and MT treatments increased the potassium content of tobacco leaves at stem-drying stage (Figure 3I). During the curing stage, the potassium content initially increased before stabilizing. Among them, the potassium content of tobacco leaves at stem-drying stage treated with EBR and MT was significantly higher than the control, which were 1.09-fold and 1.20-fold the control, respectively.

#### 2.5.6. Chlorogenic Acid

The content of chlorogenic acid in fresh tobacco leaves was decreased after EBR treatment (Figure 3J). The chlorogenic acid content of tobacco leaves increased during the curing stage. However, at yellowing stage and color fixing stage, there were no significant differences among the EBR treatment, MT treatment, and control. At stem-drying stage, the chlorogenic acid content of EBR treated tobacco leaves was significantly higher than the control and MT treatment, and its content was 1.12-fold the control. No significant difference was observed between the MT treatment and the control.

### 2.6. Transcriptome Analysis

**Figure 4:**
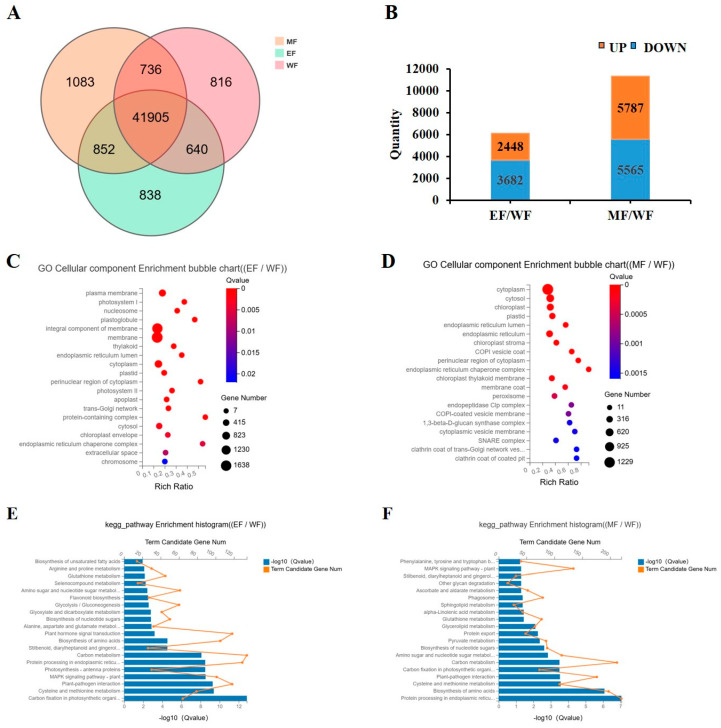
Analysis of gene expression level in transcriptome of fresh tobacco leaves. (**A**) Co-expression Venn diagram; (**B**) Number of differential genes; (**C**) Gene ontology (GO) enrichment analysis of differentially expressed genes (DEGs) in EF/WF; (**D**) GO enrichment analysis of DEGs in MF/WF; (**E**) Kyoto encyclopedia of genes and genomes (KEGG) enrichment analysis of DEGs in EF/WF; (**F**) KEGG enrichment analysis of DEGs in MF/WF. WF, fresh tobacco leaves of control; EF, fresh tobacco leaves of EBR-treated; MF, fresh tobacco leaves of MT-treated.

To investigate the effects of EBR and MT on the maturity of tobacco leaves, the transcriptome analysis was conducted on fresh tobacco leaves subjected to EBR treatment, MT treatment, and control group. A total of 9 libraries were established using the 3 varieties of samples with three biological replicates for each variety. The Q20 and Q30 values for each library were equal to or greater than 97.56 and 92.31%, respectively (Supplementary Table S2). The Venn diagram revealed that 44,235 genes were expressed in the EBR treatment, 44,576 genes in the MT treatment, and 44,097 genes in the control group. There were 41,905 genes co-expressed in all three treatments (Figure 4A). A total of 6129 differentially expressed genes (DEGs) were identified in the EBR treatment and control comparison group (EF/WF), of which 2448 genes were up-regulated and 3681 genes were down-regulated. A total of 11,352 DEGs were identified in MT treatment and control comparison group (MF/WF), of which 5787 genes were up-regulated and 5565 genes were down-regulated (Figure 4B).

To elucidate the potential function of DEGs in tobacco transcriptome following EBR and MT treatments, Gene Ontology (GO) analysis was performed on DEGs treated with EBR compared to the control, as well as DEGs treated with MT versus the control (Figure 4C,D). The results indicated that in the EF/WF GO analysis, the primary enriched pathways included membrane, integral component of membrane, photosystem I, photosystem II, plasma membrane, and chloroplast envelope. In the MF/WF GO analysis, the main enriched pathways comprised cytoplasm, cytosol, chloroplast, plastid, endoplasmic reticulum, chloroplast stroma, and chloroplast thylakoid membrane (Figure 4C,D).

To further investigate the potential functions of DEGs in tobacco transcriptome following EBR and MT treatments, Kyoto Encyclopedia of Genes and Genomes (KEGG) enrichment analysis was conducted on these DEGs (Figure 4E,F) to identify the metabolic pathways influenced by them. Based on the *p* value, the top 20 statistically significant pathways were selected to create a KEGG histogram. The results indicated that the most enriched pathways in the EF/WF KEGG analysis included carbon fixation in photosynthetic organic compounds, cysteine and methionine metabolism, plant-pathogen interactions, photosynthesis-antenna proteins, the MAPK signaling pathway in plants, carbon metabolism, biosynthesis of amino acids, and plant hormone signal transduction. In the MF/WF KEGG analysis, the most enriched pathways included protein processing in endoplasmic reticulum, biosynthesis of amino acids, cysteine and methionine metabolism, plant-pathogen interaction, carbon fixation in photosynthetic organic compounds, and carbon metabolism (Figure 4E,F).

**Figure 5:**
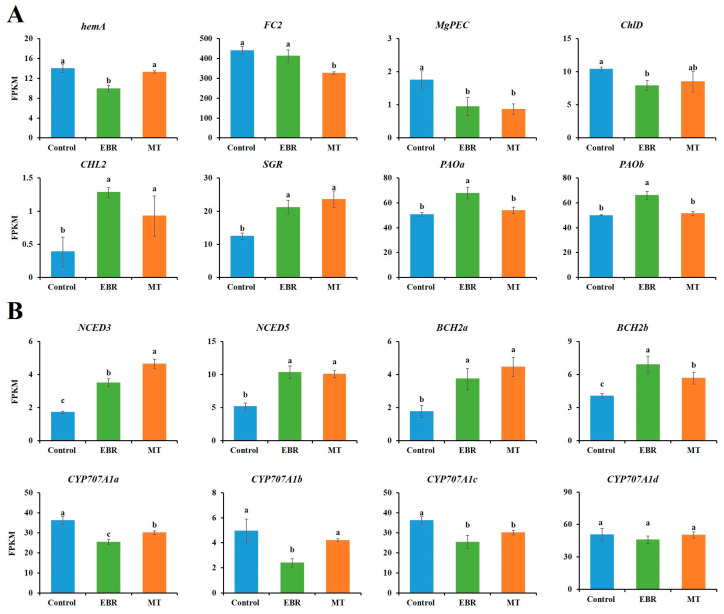
The expression of differentially expressed genes (DEGs) in pigment pathway. (**A**) The expression of DEGs in Porphyrin and chlorophyll metabolism pathway; (**B**) The expression of DEGs in Carotenoid biosynthesis pathway. EBR, tobacco leaves of EBR-treated; MT, tobacco leaves of MT-treated. “a, b, c” in the table mean significant difference among different treatments (*p* < 0.05).

Due to the significant differences in pigment content of fresh tobacco leaves at harvest, GO enrichment and KEGG enrichment analysis revealed that several differential genes were enriched in photosystem I, photosystem II, chloroplast envelope, photosynthesis-antenna proteins. We combined pigment content and transcriptome data to analyze differential genes in the chlorophyll and carotenoid biosynthetic pathways. The results indicated that genes associated with chlorophyll biosynthesis, including *hemA* (LOC107763283), MgPEC (LOC107786828), and ChlD (LOC107820629), were significantly down-regulated after EBR treatment. In contrast, chlorophyll degradation genes CHL2 (LOC107767570), SGR (LOC107817134), PAOa (LOC107805002), and PAOb (LOC107817296) were significantly up-regulated. Under MT treatment, FC2 (LOC107774460) and MgPEC were significantly down-regulated, while CHL2 and SGR were significantly up-regulated (Figure 5A).

Following EBR and MT treatments, the carotenoid degradation genes NCED3 (LOC107814412), NCED5 (LOC107822968), BCH2a (LOC107789701), and BCH2b (LOC107809222) were significantly up-regulated in both treatment groups, with the effect being more pronounced in the EBR treatment. The downstream genes of carotenoid biosynthetic pathway, such as *CYP707A1a* (*LOC107776975*), *CYP707A1b* (*LOC107804348*) and *CYP707A1c* (*LOC107830097*) were significantly down-regulated in EBR treatment, while *CYP707A1d* (*LOC107791402*) did not change significantly. In MT treatment group, *CYP707A1a* and *CYP707A1c* were significantly down-regulated, while *CYP707A1b* and *CYP707A1d* exhibited no significant changes (Figure 5B).

### 2.7. Verification of RNA-Seq Data Using qRT-PCR

To validate the accuracy and reproducibility of the RNA-Seq results, 15 DEGs were selected for qRT-PCR detection. Almost all these genes exhibited a similar expression trend in both techniques, affirming the credibility of the data obtained from RNA-Seq analysis (Supplementary Figure S1).

### 2.8. Metabolomic Analysis

**Figure 6:**
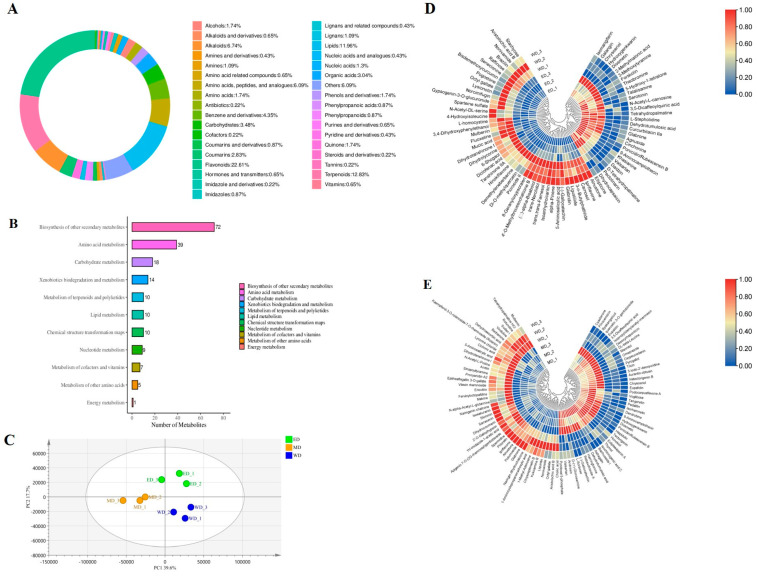
Metabolomic analysis of tobacco leaves at stem-drying stage. (**A**) Metabolite classification; (**B**) Kyoto encyclopedia of genes and genomes (KEGG) enrichment analysis of metabolites; (**C**) Partial least squares discriminant analysis (PLS-DA) plot; (**D**) Clustering analysis of differentially abundant metabolites (DAMs) in ED/WD; (**E**) Clustering analysis of DAMs in MD/WD. WD, tobacco leaves of control at stem-drying stage; ED, tobacco leaves of EBR-treated at stem-drying stage; MD, tobacco leaves of MT-treated at stem-drying stage.

To investigate the effect of exogenous spraying EBR and MT on tobacco ripening, 772 metabolites were identified by metabolomics analysis of tobacco leaves at stem-drying stage. These metabolites were classified using the KEGG and HMDB databases, with flavonoids, terpenoids, lipids, and alkaloids accounting for 22.61%, 12.83%, 11.96%, and 6.74% of the total, respectively (Figure 6A). The metabolites were predominantly enriched in the biosynthesis of other secondary metabolites, and amino acid metabolism, with 72 and 39 metabolites identified in each category, respectively (Figure 6B).

An overview of the effects of two exogenous growth regulators on tobacco leaves at stem-drying stage metabolites was provided by partial least squares discriminant analysis (PLS-DA) model analysis (Figure 6C). The differences in metabolites of tobacco leaves treated with EBR, MT, and control at stem-drying stage were characterized by PLS-DA. The first principal component (PC1) and second principal component (PC2) explained 39.6% and 17.7% of the variance, respectively. In the PLS-DA model, MT treatment and control of tobacco leaves samples were well separated along PC1, and EBR treatment and control of tobacco leaves samples were well separated along PC2. Differentially abundant metabolites (DAMs) were screened by the criteria of fold change (FC) ≥ 1.2 or ≤ 0.8, VIP ≥ 1 and p value < 0.05. Among the metabolites identified from comparison between the EBR treatment group and the control group (ED/WD), there were 74 DAMs, of which 30 were significantly up-regulated and 44 were significantly down-regulated (Figure 6D). In the comparison between the MT treatment group and the control group (MD/WD), a total of 92 DAMs were identified, of which 46 were significantly up-regulated and 46 were significantly down-regulated (Figure 6E).

**Figure 7:**
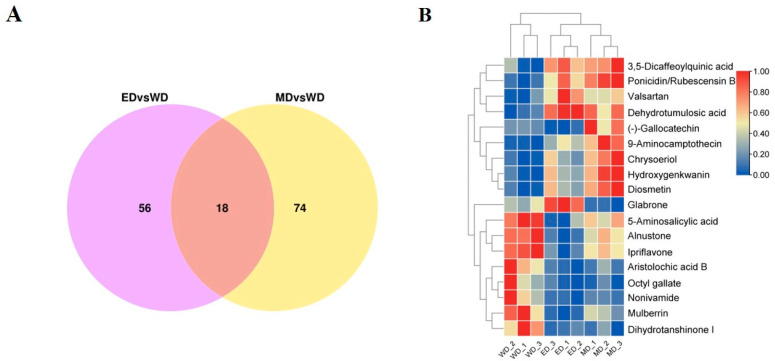
Analysis of co-expressed differentially abundant metabolites (DAMs). (**A**) Co-expression Venn diagram; (**B**) Cluster analysis of co-expressed DAMs.

To further investigate the effects of EBR and MT on the DAMs in tobacco leaves at stem-drying stage, it was found in the Venn diagram that there were 18 common DAMs of ED/WD and MD/WD (Figure 7A), of which 16 DAMs exhibited the same trend, in which 8 DAMs were up-regulated, such as diosmetin, chrysoeriol, and hydroxygenkwanin, while 8 DAMs were down-regulated, such as aristolochic acid B, octyl gallate, and 5-aminosalicylic acid. Notably, two exceptional DAMs showed contrasting regulation: glabrone was up-regulated in ED and down-regulated in MD, (-) -gallocatechin was up-regulated in MD and down-regulated in ED (Figure 7B). In addition, there were 56 DAMs unique to ED/WD and 74 DAMs unique to MD/WD.

### 2.9. Correlation Analysis

**Figure 8:**
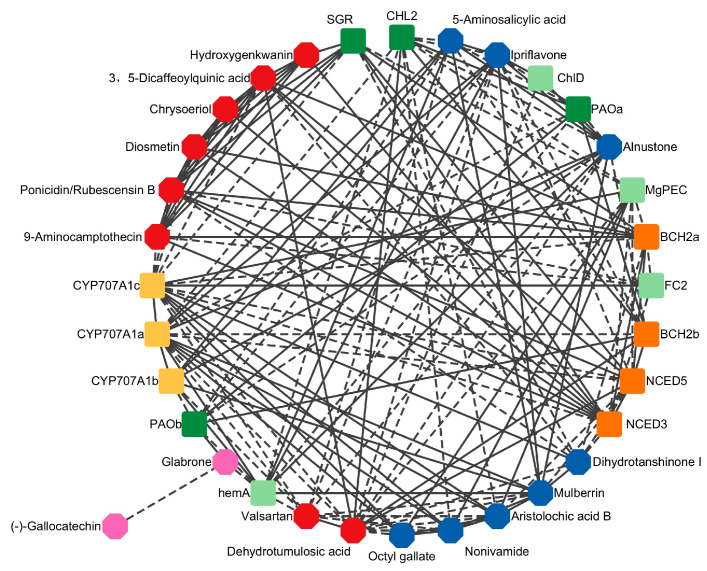
Correlation analysis between co-expressed differentially abundant metabolites (DAMs) and pigment genes. The octagonal is DAMs, the square is the pigment gene.

To investigate the correlation between differential metabolites and differential genes, we filtered Pearson correlation coefficient values (*p* > 0.80) by threshold, and used 18 DAMs, 15 DEGs and 162 lines to construct network analysis (Figure 8). The correlation network revealed that 91 groups were positively correlated and 71 groups were negatively correlated. Among them, the differential gene *CYP707A1c* exhibited the highest number of related substances and genes, 17 of which were positively correlated with 4 DAMs, 6 DAMs were negatively correlated, 3 DEGs were positively correlated, and 4 DEGs were negatively correlated. There were 16 related substances and genes of the differential metabolite dehydrotumulosic acid, which were positively correlated with 2 DAMs, 7 DAMs were negatively correlated, 3 DEGs were positively correlated, and 4 DEGs were negatively correlated. The correlation with the differential metabolite (-) -gallocatechin was the least, only negatively correlated with glabrone. The correlation between DAMs and DEGs is detailed in the electronic Supplementary Table S3.

## 3. Discussion

**Figure 9:**
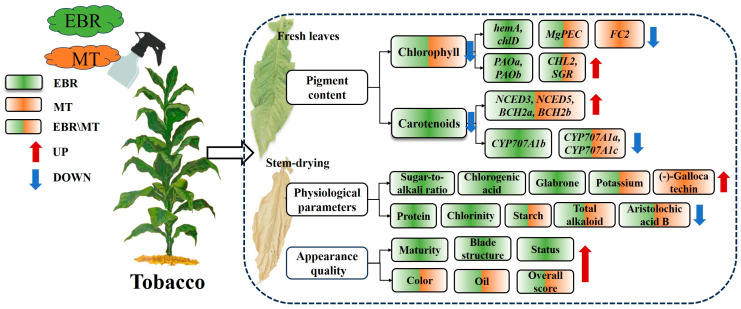
Physiological and molecular regulatory mechanisms by which exogenous 2,4-epibrassinolide and melatonin application enhances tobacco maturity and flue-cured quality. The red arrows indicate increases, while the blue arrows signify decreases in the levels of various substances and gene expression. The green box indicates the effective regulation of EBR treatment, the orange box indicates the effective regulation of MT treatment, and the green and orange mixed boxes indicate that both EBR and MT regulation are effective.

Numerous studies have demonstrated that EBR and MT play crucial roles in promoting crop growth and development, flowering, fruit ripening, and enhancing resistance to abiotic stress [16,27,28]. This study investigates the physiological and molecular mechanisms underlying the improvement of tobacco leaf quality through EBR and MT treatment. Specifically, EBR and MT treatments enhance the maturity of tobacco leaves by regulating the pigment content, as well as improving the overall quality during the stem-drying stage by influencing physiological parameters and aesthetic characteristics. Additionally, transcriptome data were employed to identify pigment-related genes responsive to EBR and MT application, thereby characterizing the molecular mechanisms by which EBR and MT enhance tobacco leaf maturity (Figure 9).

The evaluation of tobacco appearance quality is the most direct way to judge the quality and leaf grading of tobacco, and it is an important basis for determining the economic value of tobacco [29]. Previous studies have demonstrated that pre-harvest spraying of EBR has been shown to enhance the appearance quality of daylily flower buds [30] and cherry [31]. Similarly, pre-harvest spraying of MT has been shown to enhance the appearance quality of apple [32], grape [33], and strawberry [34]. In this study, the evaluation of the appearance quality of tobacco leaves at stem-drying stage following three different treatments was found that the score of EBR treatment was the highest, followed by MT treatment, and the score of control was the lowest, which indicated that EBR treatment was beneficial to enhance the appearance quality of tobacco leaves at stem-drying stage leaves, followed by MT treatment.

The content of chlorophyll and carotenoids are closely associated with the maturity of tobacco [35]. In our study, the pigment contents in tobacco leaves were significantly reduced after EBR and MT treatments, indicating that EBR and MT treatments may help to enhance the maturity of tobacco leaves. Previous studies have demonstrated that the application of BRs during the maturity stage of tomato fruit can effectively induce tomato fruit ripening and significantly reduce chlorophyll content [17]. In addition, EBR treatment significantly increased the accumulation of carotenoids and chlorophyll in Chinese kale leaves, however, this finding contrasts with our results, which may be caused by the differences of species and leaf growth stages [36]. Similarly, MT treatment was considered to have a positive regulatory effect on the ripening of grape [33], tomato [37]. and it was also found on tobacco that exogenous MT could promote the ripening and yellowing of the upper leaves of tobacco [38]. However, in our study, the content of chlorophyll a is about 1.5-fold that of chlorophyll b, which may be due to the light conditions of tobacco. The study found that low light conditions will promote the increase of chlorophyll b content [39,40], or the determination method of chlorophyll [41,42].

It was found by transcriptome analysis that the expression of chlorophyll biosynthetic genes *hemA*, *MgPEC* and *ChlD* was significantly down-regulated in EBR-treated plants, and chlorophyll biosynthesis was inhibited by their down-regulation. It has been found that *HemA*, encoding glutamyl-tRNA reductase is essential for chlorophyll biosynthesis in rice, several chlorophyll biosynthetic genes were down-regulated synchronously by the repression of *HemA* [43]. While the expression of chlorophyll decomposition genes *CHL2*, *SGR*, *PAOa* and *PAOb* was significantly up-regulated. It was found that expression of *CHL* resulted in limited chlorophyll breakdown in protoplasts by pumpkin and tobacco [44,45]. Similarly, it was found that the expression of chlorophyll biosynthetic genes *FC2* and *MgPEC* was significantly down-regulated in MT-treated plants, *FC2* was found to promote the synthesis of chlorophyll in Chinese cabbage leaves [46]. While the degradation genes *CHL2* and *SGR* were significantly up-regulated. Melatonin typically downregulates chlorophyll degradation genes, thereby slowing chlorophyll breakdown [47]. However, some studies have reported that it can accelerate color change and ripening in apples [32] and tomatoes [37]. Our study found that melatonin treatment promoted the maturation of tobacco leaves. Research on tobacco has shown that melatonin treatment promotes chlorophyll synthesis in tobacco leaves during the vegetative growth phase. However, it does not enhance chlorophyll accumulation in mature leaves, leading to excessive greening and delayed ripening [38]. Notably, the chlorophyll content in MT-treated plants was higher than that in EBR-treated plants, this difference may be attributed to the increased expression of chlorophyll decomposition genes *PAOa* and *POAb* in EBR-treated plants, whereas MT treatment had no effect on the expression levels of these genes in tobacco leaves.

In the carotenoid biosynthetic pathway, the genes *NCED3*, *NCED5*, *BCH2a*, and *BCH2b* related to carotenoid decomposition were up-regulated following EBR and MT treatment. Carotenoid decomposition could be promoted by *NCED3* and *NCED5*, while the hydroxylation of β-carotene involved in lutein biosynthesis was catalyzed by *BCH2* [48]. The regulation of abscisic acid (ABA) decomposition gene *CYP707A1s* was significantly down-regulated, which may promote the maturity of tobacco leaves. However, only the carotenoids content of EBR treated leaves decreased, which may be due to the regulation of carotenoid biosynthesis by other genes (Supplementary Figure S2). Interestingly, both EBR and MT have a significant effect on the genes downstream of the carotenoid biosynthesis pathway, increasing the expression of *NCEDs*, which can promote the synthesis of ABA [49]. The expression of *CYP707A1s* was reduced, this gene initiates ABA degradation through the oxidation of ABA to 8′-hydroxy ABA [50]. Therefore, the degradation of ABA may be alleviated, promoting the maturity of tobacco leaves.

The yellowing stage is the first and crucial step associated with phenotypic changes and physiological senescence of tobacco leaves during the curing stage. During this stage, complex physical, physiological, and biochemical reactions occur in tobacco leaves [37]. The water loss of tobacco leaves follows a pattern characterized by ‘slow in the early stage, fast in the middle stage, and slow in the later stage’ during the curing stage [51]. In our study, it was found that the water content of control decreased significantly in the early stage of curing. Furthermore, the changes in water content of tobacco leaves subjected to EBR and MT treatments adhered to this pattern and were significantly higher than those of the control group at yellowing stage. The yellowing stage of curing was the critical period for the conversion of macromolecular substances into aroma substances in tobacco leaves [52]. The study found that exogenous MT can promote the water loss rate of cigar tobacco leaves to show a ‘slow-fast-slow’ change, while mitigating the water loss rate at yellowing stage [53].

The quality and grade of tobacco are directly related to conventional chemical components [6]. Starch, the primary carbohydrate in tobacco, which can make the smoke have a mellow aroma. However, excessive starch content can negatively impact sensory quality and safety [54,55]. In our study, it was found that starch decomposition predominantly occurs during the yellowing stage of tobacco leaf curing, likely due to the high moisture content and strong amylase activity present in the leaves at this stage. Previous studies have found that prolonging the yellowing stage can promote the degradation of starch [56]. Sugars are the most abundant aroma precursors in tobacco and the main factors determining the sweetness of tobacco [3,4]. Alkaloids are the most important secondary metabolites in tobacco [1]. Nicotine accounts for more than 90% of tobacco alkaloids, reflecting the concentration of tobacco flavor. However, excessive nicotine levels increase the risk of cancer [5,57,58]. It was found that spraying EBR after tobacco topping can increase the alkaloid content of tobacco leaves in low temperature areas [59]. This contradicts our research results, which may be attributed to differences in spraying periods, temperature, and curing methods. In addition, the appropriate concentration of exogenous MT treatment can reduce the alkaloid content of tobacco leaves [60]. In our study, it was found that the sugar-to-alkali ratio increased significantly. The sugar-to-alkali ratio is an important index to measure the coordination of tobacco leaves, which can reflect the quality, safety, and sweetness of tobacco leaves [11].

The content of chlorine and potassium have important effects on the flammability, color, and ash holding capacity of tobacco [9]. Excessive chlorine content adversely affects the combustibility of tobacco leaves [61]. Conversely, higher potassium levels enhance flammability and hygroscopicity, reduce tar content, and improve the color and overall characteristics of tobacco. In the United States, the potassium content of tobacco leaves typically ranges from 4% to 6%, whereas in China, it is considerably lower, averaging between 1% and 2% [62]. It was found that exogenous EBR promotes the reduction of chlorine content and the increase of potassium content in middle and upper leaves of tobacco, corroborating our research findings [59]. Additionally, appropriate concentrations of MT can also increase potassium content [60]. Chlorogenic acid, a key polyphenol in tobacco, plays a crucial role in the color and curing characteristics of the leaves [63], as well as in the aroma quality and quantity [64,65]. In our study, it was found that the chlorogenic acid content in tobacco leaves at stem-drying stage, following EBR treatment, was higher than the control, while MT treatment appeared to have no significant effect on chlorogenic acid levels. Furthermore, research indicates that EBR can improve the browning of fresh-cut lotus root slices and color by increasing polyphenol content [66], while also maintaining the appearance quality of baby mustard [67].

The quality of tobacco leaves at stem-drying stage is influenced not only by conventional chemical components but also by small molecular metabolites produced during the curing process. Previous studies have shown that secondary metabolites, including flavonoids, alkaloids, and terpenoids, contribute to the aroma of tobacco leaves during combustion, resulting in various aromas [2,6]. In our study, it was found that there were 56 kinds of DAMs unique to ED/WD, mainly alkaloids, terpenoids, and flavonoids, while there were 74 kinds of DAMs unique to MD/WD, mainly flavonoids, which may lead to different aroma and flavor of EBR and MT treatment tobacco leaves at stem-drying stage. Among the 18 common DAMs, Aristolochic acid B, a known carcinogen, [68], was found to decrease in tobacco leaves after EBR and MT treatments, with a more significant reduction observed following EBR treatment.

In addition, glabrone and (-)-gallocatechin, both flavonoids, exhibited different trends in EBR and MT-treated tobacco leaves at stem-drying stage, potentially influencing the aroma of the leaves. Interestingly, glabrone was up-regulated in EBR-treated tobacco leaves at stem-drying stage, which was widely used to treat hyperpigmentation due to its anti-inflammatory and antioxidant properties and inhibition of melanin synthesis [69]. Conversely, (-)-gallocatechin was up-regulated in MT-treated leaves and is associated with delaying aging and memory degradation [70]. In previous study, it was found that MT can increase the concentration of (-)-catechin and (-)-gallocatechin in tea, which positively affects tea quality [71]. In addition, studies have demonstrated that the quality of tobacco leaves at the stem-drying stage is closely linked to the maturity of fresh tobacco leaves [72,73,74]. Accordingly, we examined the correlation between pigment-related genes in fresh tobacco leaves and specific metabolites at the stem-drying stage. The correlation analysis of the 18 common DAMs with pigment-related genes revealed that *NCEDs* and *CYP707A1s*, which are involved in ABA metabolism, correlated with most DAMs. Therefore, our findings suggest that pre-harvest foliar spraying with EBR and MT treatments can enhance the maturity and quality of tobacco, with EBR demonstrating superior efficacy compared to MT. This provides a robust theoretical basis for improving the maturation and quality of tobacco leaves at stem-drying stage.

## 4. Materials and Methods

### 4.1. Plant Materials and Treatments

The tobacco cultivar ‘Yunyan 87’ was planted in Fuquan Base of Guizhou Academy of Tobacco Sciences (107°52′ E, 26°68′ N) following the local high-quality tobacco production standards. Two weeks before maturity, EBR (1 μmol/L) and MT (100 μmol/L) were sprayed on the middle leaves until the front and back sides of each leaf began to drip, with an interval of 2 days, a total of 3 times. The concentration and application method of treatment were selected from previous studies [75,76].

The leaves were harvested during the standard local harvest period and subsequently cured according to the ten key temperature stabilization points for Guizhou sweet-smelly tobacco ‘442’ (Supplementary Figure S3). The water content and color difference were measured in fresh leaves (F), the end of yellowing stage (Y), the end of color fixing stage (C) and stem-drying stage (flue-cured tobacco, D), with samples collected at each stage. For each replicate, nine tobacco leaves were cut, veins were removed, and the samples were immediately frozen in liquid nitrogen. Each treatment was repeated three times. The appearance quality of tobacco leaves at stem-drying stage leaves was assessed by five experts, utilizing six evaluation indices: color, maturity, blade structure, condition, oil content, and chroma [77].

### 4.2. Chlorophyll and Carotenoids

The sample powder was extracted using acetone, and the resulting supernatant was analyzed through high-performance liquid chromatography (HPLC). For the analysis, 10 μL of the sample was separated using a mixture of isopropanol and 80% acetonitrile-water at a flow rate of 0.5 mL per minute. The linear gradient for the solvent was set up as follows: at 0 min, the concentration of solvent A was 0%, which then increased to 100% over a period of 45 min. During the analysis, absorbance was measured at wavelengths of 448 nm and 428 nm. The results were reported in terms of milligrams of content per gram of dry weight (mg g^−1^) [78].

### 4.3. Conventional Chemical Compositions

The total alkaloid, starch, total sugar, reducing sugar, protein, chlorine, potassium, and chlorogenic acid were determined by continuous flow method with reference to industry standards (YC/T 468-2013, YC/T 216-2013, YC/T 159-2019, YC/T 249-2008, YC/T 162-2002), YC/T 217-2007, YC/T 202-2006) [12].

### 4.4. Transcriptome Sequencing and Analysis

Transcriptome analysis was performed on fresh tobacco leaves harvested by EBR, MT treatment and control. The chain mRNA library was constructed and sequencing on DNBSEQ (DNBSEQ Technology) platform. The data obtained by sequencing were subjected to quality control (QC), data filtering, reads comparison, gene quantitative analysis, and analysis based on gene expression level. Differentially expressed genes between samples were screened, and gene ontology (GO) function significant enrichment analysis, kyoto encyclopedia of genes and genomes (KEGG) pathway significant enrichment analysis, and cluster analysis were performed.

### 4.5. qRT-PCR Validation

RT-qPCR was performed following the instructions of the TB Green Premix Ex TaqII (Tli RNaseH Plus) kit on a Bio-Rad iCycler thermocycler (Bio-Rad, Hercules, CA, USA). Relative gene expression levels were calculated using formula 2^−ΔΔCT^. The primers used in this study were listed in Supplementary Table S4.

### 4.6. Metabolome Sequencing and Analysis

The metabolites of EBR-treated, MT-treated and control tobacco leaves at stem-drying stage were extracted. The samples were extracted and analyzed by LC-MS. Skyline (SCIEX, University of Washington, USA) a powerful and an easy-to-use quantitation package combined with BGI-WideTarget-Library a Widely Targeted Metabolic Standards Database established by BGI was used to perform metabolite identification and quantification. Then a data matrix containing information such as metabolite identification results and quantitative results was obtained, and the table will be further processed for information analysis.

### 4.7. Statistical Analysis

The data were analyzed using one-way analysis of variance with the DPS data processing system and Origin 2024. The means were compared through the least significant difference (LSD) test at a significance level of 0.05. Principal component analysis related to the curing stage was conducted using SIMCA 14.1 software. The correlation result was performed with Cytoscape v.3.5.1 [78].

## 5. Conclusions

Exogenous applications of EBR and MT facilitated the maturation of tobacco leaves, as evidenced by a decrease in chlorophyll content. Additionally, EBR treatment further contributed to a reduction in carotenoid content. Both treatments enhanced the post-baking appearance quality of the leaves and improved the coordination of their chemical components. In conclusion, spraying EBR and MT exogenously promotes the maturation and yellowing of tobacco leaves, leading to improved overall quality. Notably, EBR treatment yielded more favorable results than MT treatment.

## Data Availability

All data supporting the findings of this study are available within the paper and within its Supplementary Materials published online.

## Supplementary Materials

The following supporting information can be downloaded at: https://www.mdpi.com/article/10.3390/plants13233266/s1.
