# Supplementary material for: Preharvest Application of Exogenous 2,4-Epibrassinolide and Melatonin Enhances the Maturity and Flue-Cured Quality of Tobacco Leaves
Source: Plants (Basel). 2024 Nov 21;13(23):3266. doi: 10.3390/plants13233266 (PMC11644396; doi:10.3390/plants13233266)
Supplement: Supplementary file 1 [file plants-13-03266-s001.zip › Supplementary Figure S2.pdf]

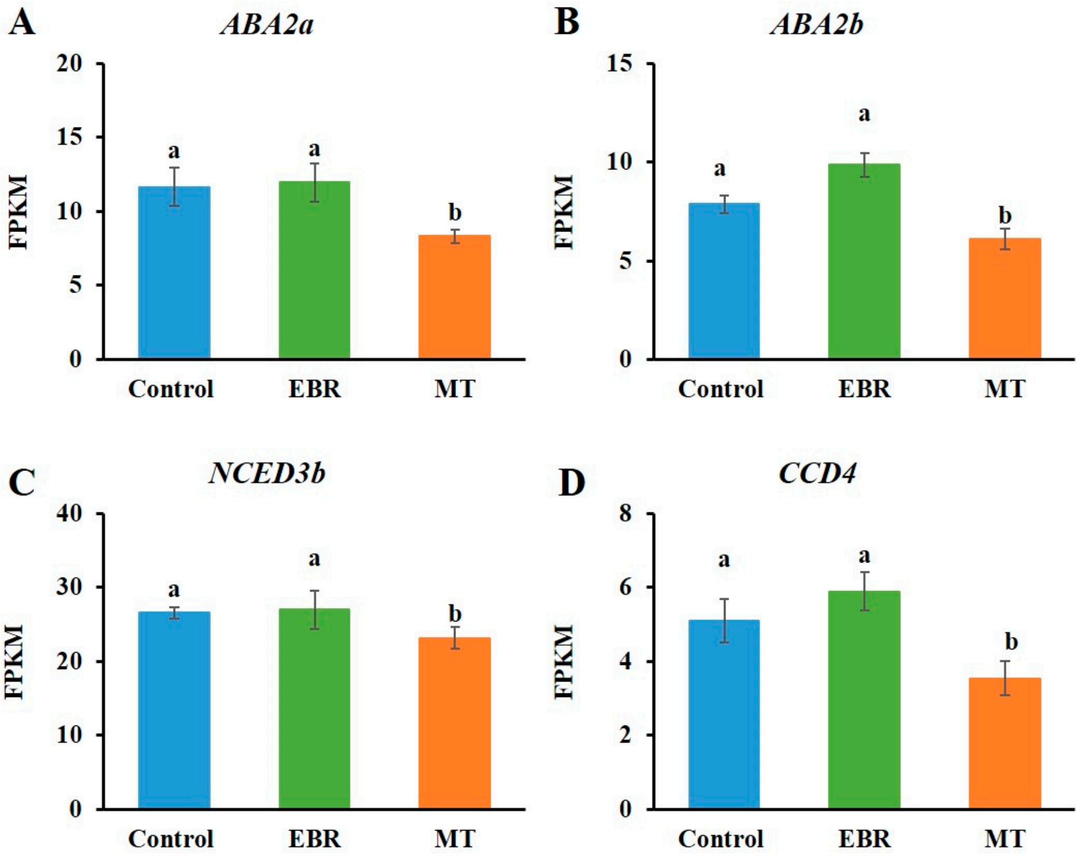

Supplementary Figure S2. The expression of differentially expressed genes (DEGs) in Carotenoid biosynthesis pathway. EBR, tobacco leaves of EBR-treated; MT, tobacco leaves of MT-treated.
