# Supplementary material for: Preharvest Application of Exogenous 2,4-Epibrassinolide and Melatonin Enhances the Maturity and Flue-Cured Quality of Tobacco Leaves
Source: Plants (Basel). 2024 Nov 21;13(23):3266. doi: 10.3390/plants13233266 (PMC11644396; doi:10.3390/plants13233266)
Supplement: Supplementary file 1 [file plants-13-03266-s001.zip › Supplementary Table S1.pdf]

Supplementary Table S1. Chromatic aberration of tobacco leaves during curing stage. F, fresh leaves; Y, yellowing stage; C, color fixing stage; D, stem-drying stage. “a, b, c” in the table mean significant difference among different treatments ($p < 0.05$).

		Chromatic aberration			
Stage		F	Y	C	D
L^*	CK	50.08a	66.07a	54.83b	63.27ab
	EBR	51.42a	64.78a	57.26b	62.58b
	MT	45.32b	64.37a	61.00a	64.13a
a^*	CK	-8.82b	8.73a	12.47a	11.36ab
	EBR	-7.66a	7.34a	10.93a	11.79a
	MT	-8.46b	8.18a	11.49a	10.51b
b^*	CK	34.13b	49.01a	46.02a	46.36b
	EBR	38.79a	43.99b	45.77a	48.66a
	MT	30.87c	47ab	46.53a	47.97ab
