# Supplementary material for: Preharvest Application of Exogenous 2,4-Epibrassinolide and Melatonin Enhances the Maturity and Flue-Cured Quality of Tobacco Leaves
Source: Plants (Basel). 2024 Nov 21;13(23):3266. doi: 10.3390/plants13233266 (PMC11644396; doi:10.3390/plants13233266)
Supplement: Supplementary file 1 [file plants-13-03266-s001.zip › Supplementary Table S2.pdf]

Supplementary Table S2. Overview of the transcriptome sequencing data and quality check.

Sample	Total Raw Reads (M)	Total Clean Reads (M)	Total Clean Bases (Gb)	Clean Reads Q20 (%)	Clean Reads Q30 (%)	Clean Reads Ratio (%)
**EF_1**	45.44	43.87	6.58	97.66	92.62	96.54
**EF_2**	45.44	43.85	6.58	98.09	93.91	96.5
**EF_3**	41.27	39.34	5.9	97.84	93.09	95.32
**MF_1**	45.44	44.31	6.65	97.56	92.31	97.51
**MF_2**	47.19	45.06	6.76	98.21	94.28	95.49
**MF_3**	45.44	43.85	6.58	98.14	94.07	96.5
**WF_1**	45.44	44.12	6.62	97.59	92.45	97.1
**WF_2**	45.44	44.15	6.62	98.07	93.8	97.16
**WF_3**	47.19	44.92	6.74	98.5	95.19	95.19
