# Supplementary material for: Preharvest Application of Exogenous 2,4-Epibrassinolide and Melatonin Enhances the Maturity and Flue-Cured Quality of Tobacco Leaves
Source: Plants (Basel). 2024 Nov 21;13(23):3266. doi: 10.3390/plants13233266 (PMC11644396; doi:10.3390/plants13233266)
Supplement: Supplementary file 1 [file plants-13-03266-s001.zip › Supplementary Table S4.pdf]

Supplementary Table S4 Primer used in this study.

Gene ID	Fprimer	Rprimer
LOC107763283	TAGTTCAGCTGCTGTGGAGC	CGACCATCCTAGTGCATCCC
LOC107774460	TCACCGAGGAAGCTGTTCAC	AGCCTTGTCGTTGGTACCAG
LOC107820629	AACCCACCAGACCAGCAAAA	TCCACTAAACCACCTTCCGC
LOC107817134	CTTCTGCAAGGAACTCCCCG	TTTGAGTTGGCCCCAGCAAT
LOC107805002	TTCACCTACCCCGTTTCAGC	CAAGGCGATGAGGGCATTTG
LOC107817296	AAGCCTGAGTTTGCGACAGT	GGGCTTGGCTCTATCTCGTC
LOC107776975	CCAGAGGCAGCAAAGCTAGT	GGATTCGGGCATGAAAGCAC
LOC107830097	ATTTGGCAATGGGGTCCACT	GGCCCATCATAGACCACCTG
LOC107827644	TCACGCCGTCCAATTCAAGA	ATGAGCCTTGCAATTCCCGA
LOC107822968	TTTGAAGACCGGCGAATCGA	CTGACACTTTTGGCCATGGC
LOC107809222	TCTCTCAGTTGGTGCTGCTG	GTTTGTGGTGTGACTCGTGC
LOC107805115	CGAGGACGGATGGATATGCA	TGTTGGAACAGAGGGTGTGT
LOC107782328	TTGATCAAACGCCGAGGGAA	GCTCCCCCATGTCCATCAAA
LOC107778431	GGACATGGCGAAAACTCAGC	TGTGCAAATGGAGGCGTTTG
LOC107820266	CAGAGGCTAACCGGCAAGAA	TCGGACTTGGTTTACACGCA
