# Supplementary figures and images for: Preharvest Application of Exogenous 2,4-Epibrassinolide and Melatonin Enhances the Maturity and Flue-Cured Quality of Tobacco Leaves
Source: Plants (Basel). 2024 Nov 21;13(23):3266. doi: 10.3390/plants13233266 (PMC11644396; doi:10.3390/plants13233266)

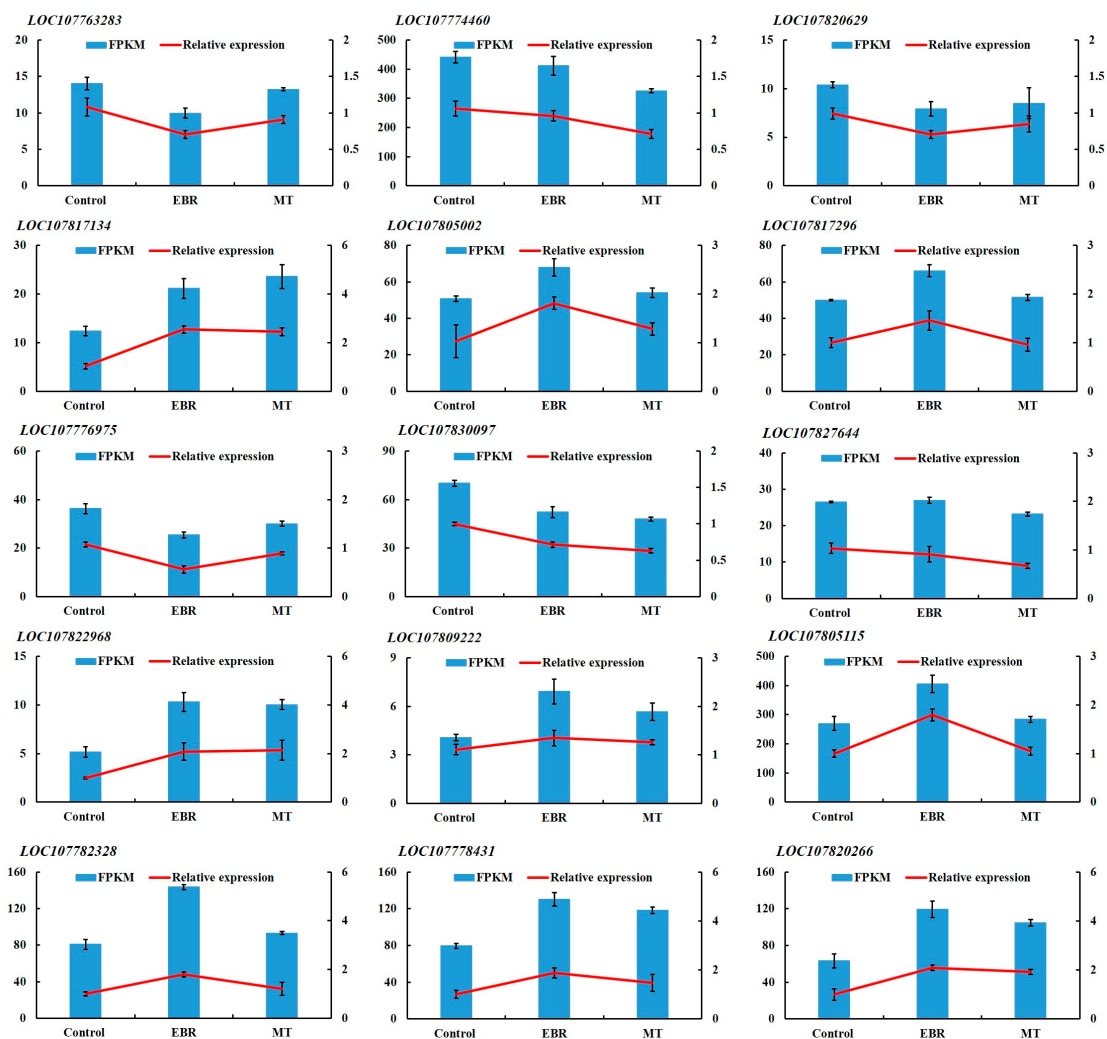

Supplementary Figure S1. Expression pattern of 15 selected DEGs obtained by RNA-Seq and qRT-PCR.

Supplement: Supplementary file 1 [file plants-13-03266-s001.zip › Supplementary Figure S1.pdf]

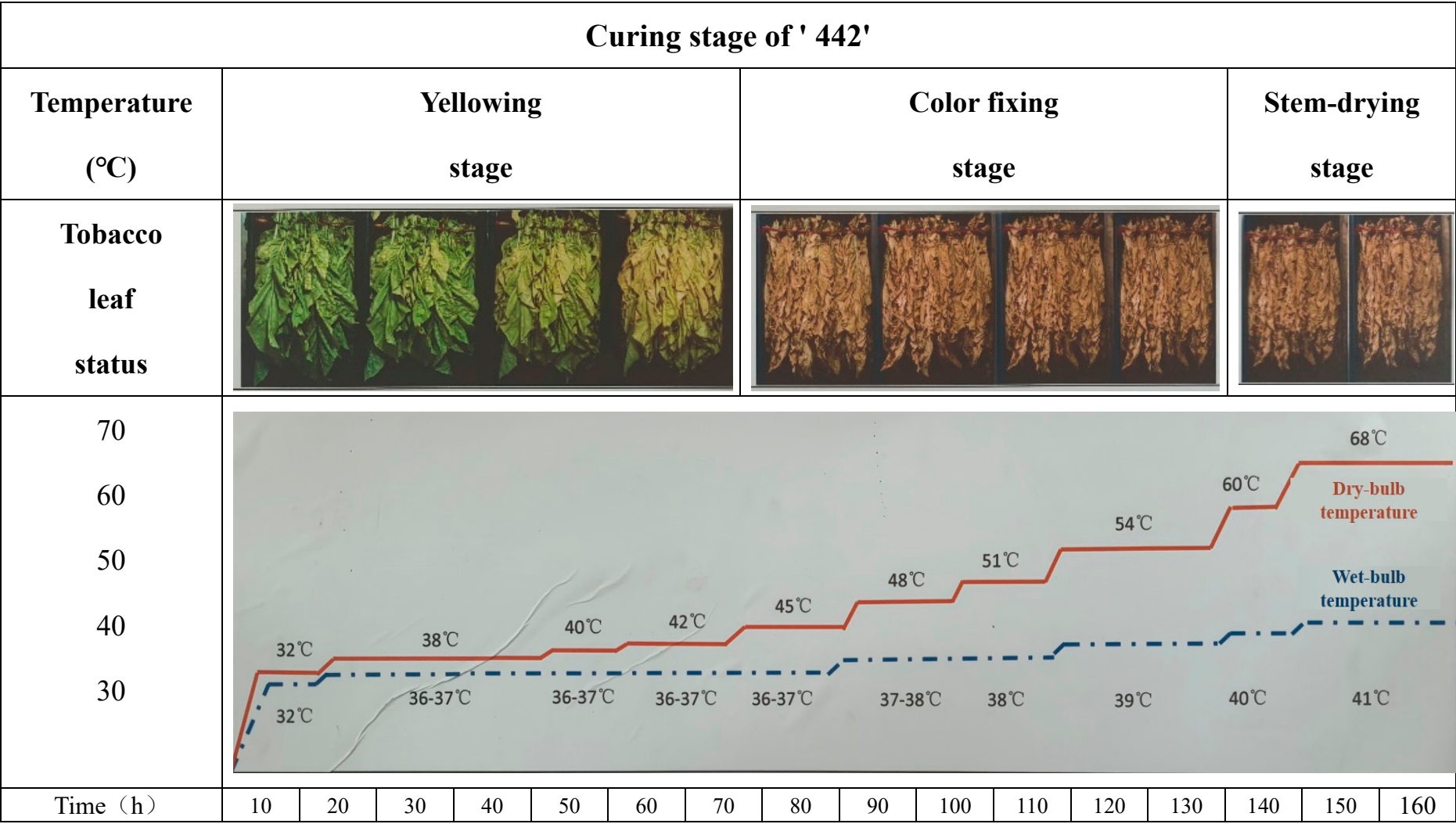

Supplementary Figure S3. Curing stage of ten key stable temperature points of '442'.

Supplement: Supplementary file 1 [file plants-13-03266-s001.zip › Supplementary Figure S3.pdf]
